# Ba_4_GaN_3_O

**DOI:** 10.1107/S1600536814010472

**Published:** 2014-05-17

**Authors:** Takayuki Hashimoto, Hisanori Yamane

**Affiliations:** a Institute of Multidisciplinary Research for Advanced Materials, Tohoku University, 2-1-1 Katahira, Aoba-ku, Sendai, 980-8577, Japan

## Abstract

Red transparant platelet-shaped single crystals of tetra­barium gallium trinitride oxide, Ba_4_GaN_3_O, were synthesized by the Na flux method. The crystal structure is isotypic with Sr_4_GaN_3_O, containing isolated triangular [GaN_3_]^6−^ anionic groups. O^2−^ atoms are inserted between the slabs of [Ba_4_GaN_3_]^2+^, in which the [GaN_3_]^6−^ groups are surrounded by Ba^2+^ atoms.

## Related literature   

For isotypic Sr_4_GaN_3_O, see: Mallinson *et al.* (2006 ▶). For the major phase in the product, Ba_3_Ga_2_N_4_, see: Yamane & DiSalvo (1996 ▶). For compounds containing isolated triangular-planar [GaN_3_]^6−^ nitridogallate anions, see: Park *et al.* (2003 ▶); Mallinson *et al.* (2006 ▶); Hintze & Schnick (2010 ▶). For details of Madelung site potential and energy calculations, see: O’Keeffe (1992 ▶); Orhan *et al.*. (2002 ▶); Paszkowicz *et al.* (2004 ▶); Taylor (1984 ▶). For details of the synthetic procedure, see: Kowach *et al.* (1998 ▶).

## Experimental   

### 

#### Crystal data  

Ba_4_GaN_3_O
*M* *_r_* = 677.11
Orthorhombic, 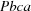

*a* = 7.8130 (3) Å
*b* = 25.6453 (10) Å
*c* = 7.9162 (4) Å
*V* = 1586.14 (12) Å^3^
*Z* = 8
Mo *K*α radiation
μ = 22.84 mm^−1^
*T* = 293 K
0.18 × 0.13 × 0.07 mm

#### Data collection  

Rigaku R-AXIS RAPID II diffractometer
Absorption correction: numerical (*NUMABS*; Higashi, 1999 ▶) *T*_min_ = 0.071, *T*_max_ = 0.411
14273 measured reflections
1820 independent reflections
1588 reflections with *I* > 2σ(*I*)
*R*_int_ = 0.133

#### Refinement  

*R*[*F* ^2^ > 2σ(*F* ^2^)] = 0.043
*wR*(*F* ^2^) = 0.099
*S* = 1.05
1820 reflections
82 parameters
Δρ_max_ = 2.54 e Å^−3^
Δρ_min_ = −1.72 e Å^−3^

### 

Data collection: *RAPID-AUTO* (Rigaku Corporation, 2005 ▶); cell refinement: *RAPID-AUTO*; data reduction: *RAPID-AUTO*; program(s) used to solve structure: *SHELXS97* (Sheldrick, 2008 ▶); program(s) used to refine structure: *SHELXL97* (Sheldrick, 2008 ▶); molecular graphics: *VESTA* (Momma & Izumi, 2008 ▶); software used to prepare material for publication: *SHELXL97*.

## Supplementary Material

Crystal structure: contains datablock(s) I. DOI: 10.1107/S1600536814010472/hp2067sup1.cif

Structure factors: contains datablock(s) I. DOI: 10.1107/S1600536814010472/hp2067Isup2.hkl

Supporting information file. DOI: 10.1107/S1600536814010472/hp2067Isup3.cml

CCDC reference: 1001671

Additional supporting information:  crystallographic information; 3D view; checkCIF report

## Figures and Tables

**Table:** 

Ba1—N1^i^	2.675 (8)
Ba1—N1	2.799 (9)
Ba1—N2^ii^	2.998 (7)
Ba1—O1^iii^	2.999 (8)
Ba1—O1^iv^	3.054 (9)
Ba2—O1^v^	2.683 (9)
Ba2—N2^v^	2.687 (8)
Ba2—N1^iii^	2.991 (9)
Ba2—N2	3.158 (8)
Ba2—O1	3.184 (9)
Ba2—O1^vi^	3.264 (10)
Ba3—N3^vii^	2.730 (7)
Ba3—N3^i^	2.762 (8)
Ba3—N3	2.764 (7)
Ba3—N1^i^	2.914 (9)
Ba3—N3^ii^	2.994 (8)
Ba4—N3^ii^	2.661 (8)
Ba4—N2	2.808 (7)
Ba4—N2^v^	2.862 (8)
Ba4—O1	3.133 (10)
Ba4—N1^viii^	3.231 (9)
Ga1—N3	1.876 (8)
Ga1—N2	1.908 (8)
Ga1—N1^iii^	1.924 (8)

**Table:** 

N3—Ga1—N2	125.3 (3)
N3—Ga1—N1^iii^	119.8 (3)
N2—Ga1—N1^iii^	114.6 (4)

Symmetry codes: (i) 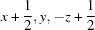; (ii) 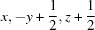; (iii) 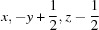; (iv) 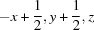; (v) 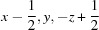; (vi) 
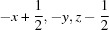
; (vii) 
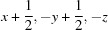
; (viii) 
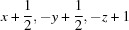
.

**Fig. 1.**
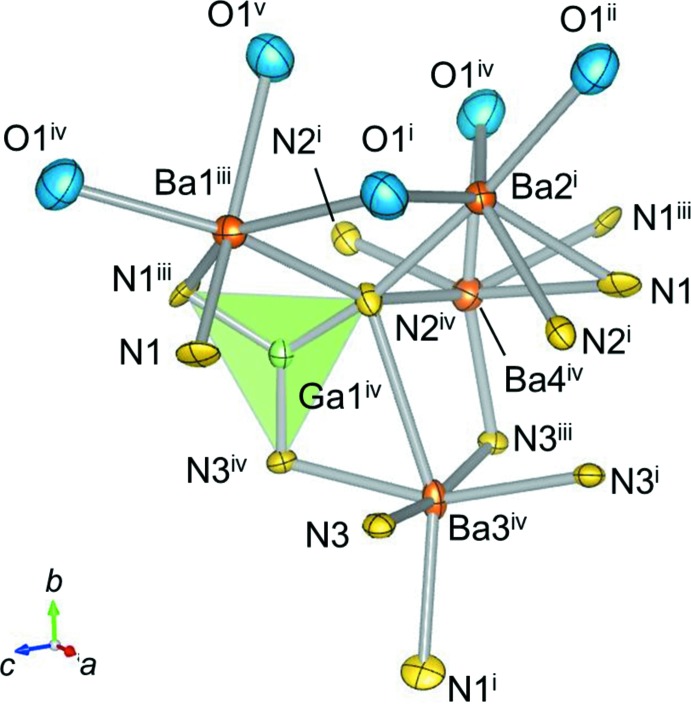
The atomic arrangement around Ba and Ga atoms in the structure of Ba4GaN3O. Displacement ellipsoids are drawn at 70% probability. Symmetry codes: (i) x, -y + 1/2, z + 1/2; (ii) -x + 1/2, y + 1/2, z; (iii) x + 1/2, y, -z + 1/2; (iv) x + 1/2, -y + 1/2, -z; (v) -x, y + 1/2, -z + 1/2.

**Fig. 2.**
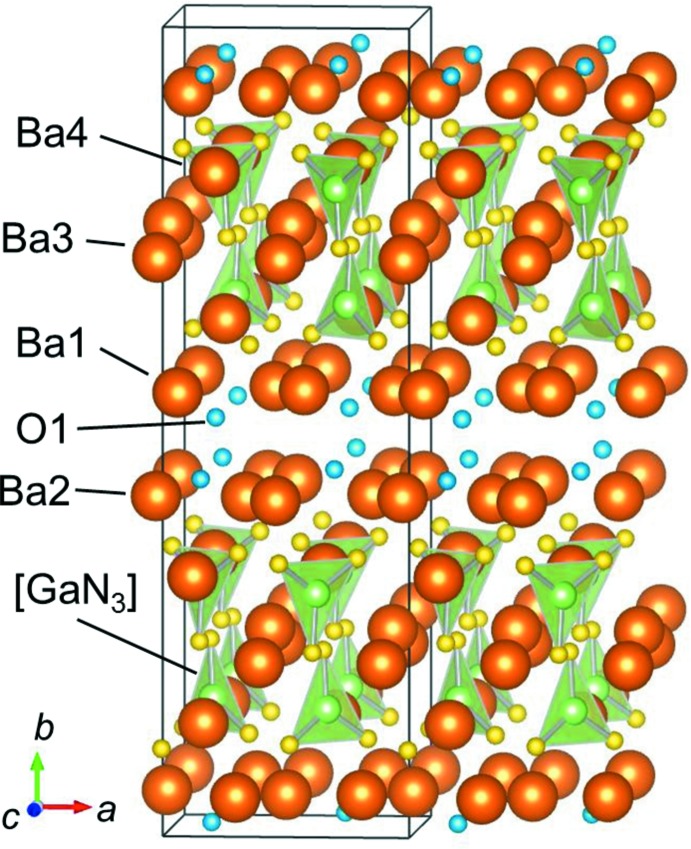
Crystal structure of Ba4GaN3O illustrated with Ga-centered N atom triangles.

## supplementary crystallographic information

### 1. Comment

Ba_4_GaN_3_O is isostructural with Sr_4_GaN_3_O (Mallinson *et al.*, 2006) which crystallizes in an orthorhombic cell with the space group *Pbca* (No. 61). The coordination environment around Ga1 site and Ba1–Ba3 sites are shown in Fig.1. Ga1 atom is bonded to N1, N2 and N3 atoms and form a triangular anionic group of [GaN_3_]^6-^. Ga—N bond lengths of 1.876 (8)–1.924 (8)Å are comparable with those observed in [GaN_3_]^6-^ groups of Sr_3_GaN_3_ and Sr_6_GaN_5_ (1.938 Å, 1.895 Å, Park *et al.*, 2003), Sr_4_GaN_3_O (1.880–1.921 Å, Mallinson *et al.*, 2006) and LiBa_5_GaN_3_F_5_ (1.896–1.945 Å, Hintze & Schnick, 2010). Ba1 atom is coordinated by two N1, one N2 and three O1 atoms, and Ba2 atom is by one N1, two N2 and three O1 atoms. N1 and N2 atoms are in seven-fold coordination sites of one Ga and six Ba atoms, and N3 atom is in the six-fold coordination site of one Ga and five Ba atoms. O1 atom is coordinated by seven Ba atoms. As shown in Fig. 2, O1 atoms are situated at the sites between [Ba_4_GaN_3_] slabs which are composed of triangular [GaN_3_] groups and Ba atoms in the *a*–*c* plane.

Mallinson *et al.*, (2006) calculated Madelung site potential and Madelung energy per formula of Sr_4_GaN_3_O for four models of O and N atom arrangement. They concluded the model with O atom located at the O1 site coordinated by only Sr atoms is the most stable structure because this model showed the smallest deviation of the site potentials in atom sites of the same species and the lowest energy. The site potentials and energy calculated by using *EUTAX* (O'Keeffe, 1992) and *VESTA* (Momma & Izumi, 2008) programs with the data of the present study are -17.12 – -17.87 V for Ba1–Ba4, -36.17 V for Ga1, 28.24 – 29.45 V for N1–N3, 16.86 V for O1, and -26,100 kJ/mol for Ba_4_GaN_3_O. The values of Ga, N and O sites were consistent with the site potentials reported for Sr_4_GaN_3_O (Ga: -35.01 V, N: 29.58 – 31.38 V, O: 17.36 V) (Mallinson *et al.*, 2006). The difference between the Madelung energy per formula of Ba_4_GaN_3_O and the sum of Madelung energies of Ba_3_N_2_ derived by the theoretical calculation (-12,200 kJ/mol, Orhan *et al.*, 2002), BaO (-3,500 kJ/mol, Taylor, 1984) and GaN (-10,500 kJ/mol, Paszkowicz *et al.*, 2004) calculated from the crystal structure data is 0.4%.

### 2. Experimental

Starting materials were pieces of Ba (Sigma-Aldrich, 99.99%), Ga (Rasa Industries, 99.99995%) and Na (Nippon Soda Co. Ltd., 99.95%), and powders of Si (Kojundo Chemical Laboratory, 99.999%) and NaN_3_ (Toyo Kasei Kogyo Co. Ltd., 99.9%). In an Ar gas-filled glove box (O_2_ < 1 ppm, H_2_O < 1 ppm), Ba (1.00 mmol), Ga (0.25 mmol), Na (2.4 mmol), Si (0.50 mmol) and NaN_3_ (1.2 mmol) were weighed and placed in a BN crucible (Showa Denko, 99.5%). The crucible was sealed in a stainless-steel tube. The sample was heated to 750°C in an electric furnace with a rate of 6°C min^-1^. This temperature was maintained for 2 hours and lowered to 550°C with a cooling rate of -2.8°C min^-1^. After that the sample was cooled to room temperature by shutting off the electric power to the furnace. The stainless-steel tube was cut and opened in the glove box, and the crucible was washed with liquid NH_3_ (Japan Fine Products, >99.999%) to dissolve away Na. The details of the Na removing method have been described in the literature (Kowach *et al.*, 1998). The initial objective was to synthesize a Ba–Ga–Si–N quaternary compound by the Na flux method, but the main product obtained was yellow transparent granular single crystals of Ba_3_Ga_2_N_4_ (Yamane & DiSalvo, 1996). A small amount of red transparent platelet single crystals of Ba_4_GaN_3_O were included in the product.

Semi-qu­anti­tative elemental analysis of the red single crystals was carried out with an energy-dispersive X-ray detector (EDX, EDAX, Genesis) attached to a scanning electron microscope (SEM, Hitachi, S-4800). Ba:Ga molar ratio determined by the EDX analysis was 78:22 which was close to the ratio (4:1) of Ba_4_GaN_3_O. The oxygen was probably originated from the surface oxide layers of the starting materials. Since Ba_4_GaN_3_O is unstable in air, a single crystal was picked up from the product and sealed in glass capillaries in the glove box for XRD data collection.

The peaks of 2.25–2.54 e Å^-3^ in the *F*_o_–*F*_c_ map were observed at 0.88–0.96 Å distant from Ba1–Ba3 atoms. These large differences are probably a result of the cut-off effect of the Fourier synthesis.

### Crystal data

**Table:**

Ba_4_GaN_3_O	*F*(000) = 2272
*M**_r_* = 677.11	*D*_x_ = 5.671 Mg m^−^^3^
Orthorhombic, *P**b**c**a*	Mo *K*α radiation, λ = 0.71075 Å
Hall symbol: -P 2ac 2ab	Cell parameters from 11112 reflections
*a* = 7.8130 (3) Å	θ = 3.0–27.5°
*b* = 25.6453 (10) Å	µ = 22.84 mm^−^^1^
*c* = 7.9162 (4) Å	*T* = 293 K
*V* = 1586.14 (12) Å^3^	Platelet, red
*Z* = 8	0.18 × 0.13 × 0.07 mm

### Data collection

**Table:**

Rigaku R-AXIS RAPID II diffractometer	1820 independent reflections
Radiation source: fine-focus sealed tube	1588 reflections with *I* > 2σ(*I*)
Graphite monochromator	*R*_int_ = 0.133
Detector resolution: 10.0 pixels mm^-1^	θ_max_ = 27.5°, θ_min_ = 3.0°
ω scans	*h* = −9→10
Absorption correction: numerical (*NUMABS*; Higashi, 1999)	*k* = −33→33
*T*_min_ = 0.071, *T*_max_ = 0.411	*l* = −10→10
14273 measured reflections	

### Refinement

**Table:**

Refinement on *F*^2^	0 restraints
Least-squares matrix: full	Primary atom site location: structure-invariant direct methods
*R*[*F*^2^ > 2σ(*F*^2^)] = 0.043	Secondary atom site location: difference Fourier map
*wR*(*F*^2^) = 0.099	*w* = 1/[σ^2^(*F*_o_^2^) + (0.*P*)^2^ + 18.6031*P*] where *P* = (*F*_o_^2^ + 2*F*_c_^2^)/3
*S* = 1.05	(Δ/σ)_max_ = 0.001
1820 reflections	Δρ_max_ = 2.54 e Å^−^^3^
82 parameters	Δρ_min_ = −1.72 e Å^−^^3^

### Special details

**Table:**

Geometry. All e.s.d.'s (except the e.s.d. in the dihedral angle between two l.s. planes) are estimated using the full covariance matrix. The cell e.s.d.'s are taken into account individually in the estimation of e.s.d.'s in distances, angles and torsion angles; correlations between e.s.d.'s in cell parameters are only used when they are defined by crystal symmetry. An approximate (isotropic) treatment of cell e.s.d.'s is used for estimating e.s.d.'s involving l.s. planes.
Refinement. Refinement of *F*^2^ against ALL reflections. The weighted *R*-factor *wR* and goodness of fit *S* are based on *F*^2^, conventional *R*-factors *R* are based on *F*, with *F* set to zero for negative *F*^2^. The threshold expression of *F*^2^ > σ(*F*^2^) is used only for calculating *R*-factors(gt) *etc*. and is not relevant to the choice of reflections for refinement. *R*-factors based on *F*^2^ are statistically about twice as large as those based on *F*, and *R*- factors based on ALL data will be even larger.

### Fractional atomic coordinates and isotropic or equivalent isotropic displacement parameters (Å^2^)

**Table:**

	*x*	*y*	*z*	*U*_iso_*/*U*_eq_	
Ba1	0.36690 (8)	0.42906 (2)	0.32613 (7)	0.02148 (18)	
Ba2	0.03129 (8)	0.06166 (2)	0.16149 (7)	0.02055 (18)	
Ba3	0.45621 (7)	0.26486 (2)	0.23802 (7)	0.02003 (18)	
Ba4	0.20527 (8)	0.15269 (2)	0.46083 (7)	0.02310 (19)	
Ga1	0.20637 (12)	0.17178 (4)	0.01682 (12)	0.0157 (2)	
N1	0.0710 (11)	0.3685 (3)	0.3614 (11)	0.0246 (19)	
N2	0.3630 (10)	0.1319 (3)	0.1492 (9)	0.0187 (17)	
N3	0.1943 (10)	0.2448 (3)	0.0124 (9)	0.0169 (17)	
O1	0.2416 (12)	0.0314 (4)	0.4918 (11)	0.049 (2)	

### Atomic displacement parameters (Å^2^)

**Table:**

	*U*^11^	*U*^22^	*U*^33^	*U*^12^	*U*^13^	*U*^23^
Ba1	0.0234 (3)	0.0214 (4)	0.0196 (3)	0.0002 (2)	0.0046 (2)	0.0002 (2)
Ba2	0.0211 (3)	0.0211 (4)	0.0194 (3)	−0.0001 (2)	0.0009 (2)	−0.0004 (2)
Ba3	0.0152 (3)	0.0326 (4)	0.0123 (3)	0.0000 (2)	0.0001 (2)	0.0007 (2)
Ba4	0.0258 (3)	0.0226 (4)	0.0209 (3)	0.0000 (2)	0.0001 (2)	−0.0023 (2)
Ga1	0.0158 (5)	0.0183 (6)	0.0132 (5)	−0.0002 (4)	−0.0006 (4)	−0.0001 (4)
N1	0.026 (4)	0.019 (5)	0.029 (5)	0.011 (4)	−0.010 (4)	0.006 (3)
N2	0.022 (4)	0.020 (4)	0.015 (4)	0.006 (3)	−0.002 (3)	0.000 (3)
N3	0.022 (4)	0.011 (4)	0.018 (4)	−0.002 (3)	−0.002 (3)	−0.001 (3)
O1	0.053 (5)	0.052 (6)	0.041 (5)	−0.007 (5)	0.001 (4)	−0.010 (4)

### Geometric parameters (Å, º)

**Table:**

Ba1—N1^i^	2.675 (8)	Ba4—N1^viii^	3.231 (9)
Ba1—N1	2.799 (9)	Ga1—N3	1.876 (8)
Ba1—N2^ii^	2.998 (7)	Ga1—N2	1.908 (8)
Ba1—O1^iii^	2.999 (8)	Ga1—N1^iii^	1.924 (8)
Ba1—O1^iv^	3.054 (9)	Ga1—Ba3^ix^	3.2450 (11)
Ba1—Ga1^ii^	3.2466 (12)	Ga1—Ba1^iii^	3.2467 (12)
Ba2—O1^v^	2.683 (9)	Ga1—Ba3^iii^	3.3648 (11)
Ba2—N2^v^	2.687 (8)	N1—Ga1^ii^	1.924 (8)
Ba2—N1^iii^	2.991 (9)	N1—Ba1^v^	2.675 (8)
Ba2—N2	3.158 (8)	N1—Ba3^v^	2.914 (9)
Ba2—O1	3.184 (9)	N1—Ba2^ii^	2.991 (9)
Ba2—O1^vi^	3.264 (10)	N1—Ba4^x^	3.231 (9)
Ba2—Ga1	3.3405 (12)	N2—Ba2^i^	2.687 (8)
Ba3—N3^vii^	2.730 (7)	N2—Ba4^i^	2.862 (8)
Ba3—N3^i^	2.762 (8)	N2—Ba1^iii^	2.998 (7)
Ba3—N3	2.764 (7)	N3—Ba4^iii^	2.661 (8)
Ba3—N1^i^	2.914 (9)	N3—Ba3^ix^	2.730 (7)
Ba3—N3^ii^	2.994 (8)	N3—Ba3^v^	2.762 (8)
Ba3—Ga1^vii^	3.2450 (11)	N3—Ba3^iii^	2.994 (8)
Ba3—Ga1^ii^	3.3647 (11)	O1—Ba2^i^	2.683 (9)
Ba4—N3^ii^	2.661 (8)	O1—Ba1^ii^	2.999 (8)
Ba4—N2	2.808 (7)	O1—Ba1^xi^	3.054 (9)
Ba4—N2^v^	2.862 (8)	O1—Ba2^xii^	3.264 (10)
Ba4—O1	3.133 (10)		
			
N1^i^—Ba1—N1	103.0 (3)	Ba4^iii^—Ba3—Ba4^viii^	109.76 (2)
N1^i^—Ba1—N2^ii^	100.2 (2)	Ga1^i^—Ba3—Ba4^viii^	75.61 (2)
N1—Ba1—N2^ii^	67.5 (2)	N3^vii^—Ba3—Ba4^i^	44.25 (16)
N1^i^—Ba1—O1^iii^	84.3 (3)	N3^i^—Ba3—Ba4^i^	79.12 (16)
N1—Ba1—O1^iii^	90.3 (2)	N3—Ba3—Ba4^i^	88.33 (16)
N2^ii^—Ba1—O1^iii^	157.8 (2)	N1^i^—Ba3—Ba4^i^	114.81 (15)
N1^i^—Ba1—O1^iv^	154.5 (3)	N3^ii^—Ba3—Ba4^i^	125.84 (15)
N1—Ba1—O1^iv^	101.8 (3)	Ga1^vii^—Ba3—Ba4^i^	79.26 (2)
N2^ii^—Ba1—O1^iv^	94.5 (2)	Ga1^ii^—Ba3—Ba4^i^	158.61 (3)
O1^iii^—Ba1—O1^iv^	89.88 (12)	Ga1—Ba3—Ba4^i^	64.48 (2)
N1^i^—Ba1—Ga1^ii^	91.49 (19)	Ba4^iii^—Ba3—Ba4^i^	117.798 (17)
N1—Ba1—Ga1^ii^	36.16 (16)	Ga1^i^—Ba3—Ba4^i^	56.77 (2)
N2^ii^—Ba1—Ga1^ii^	35.30 (15)	Ba4^viii^—Ba3—Ba4^i^	115.14 (2)
O1^iii^—Ba1—Ga1^ii^	123.61 (19)	N3^ii^—Ba4—N2	109.6 (2)
O1^iv^—Ba1—Ga1^ii^	112.20 (18)	N3^ii^—Ba4—N2^v^	101.6 (2)
N1^i^—Ba1—Ba2^iv^	102.02 (19)	N2—Ba4—N2^v^	96.16 (14)
N1—Ba1—Ba2^iv^	135.83 (16)	N3^ii^—Ba4—O1	166.3 (2)
N2^ii^—Ba1—Ba2^iv^	140.93 (15)	N2—Ba4—O1	80.8 (2)
O1^iii^—Ba1—Ba2^iv^	56.82 (19)	N2^v^—Ba4—O1	85.6 (2)
O1^iv^—Ba1—Ba2^iv^	54.92 (18)	N3^ii^—Ba4—N1^viii^	97.3 (2)
Ga1^ii^—Ba1—Ba2^iv^	166.34 (3)	N2—Ba4—N1^viii^	87.9 (2)
N1^i^—Ba1—Ba2^ii^	148.09 (19)	N2^v^—Ba4—N1^viii^	158.0 (2)
N1—Ba1—Ba2^ii^	52.06 (18)	O1—Ba4—N1^viii^	73.7 (2)
N2^ii^—Ba1—Ba2^ii^	54.58 (15)	N3^ii^—Ba4—Ga1	90.91 (16)
O1^iii^—Ba1—Ba2^ii^	112.10 (18)	N2—Ba4—Ga1	32.35 (15)
O1^iv^—Ba1—Ba2^ii^	56.35 (18)	N2^v^—Ba4—Ga1	74.12 (15)
Ga1^ii^—Ba1—Ba2^ii^	56.61 (2)	O1—Ba4—Ga1	102.37 (15)
Ba2^iv^—Ba1—Ba2^ii^	109.870 (18)	N1^viii^—Ba4—Ga1	116.90 (14)
N1^i^—Ba1—Ba4^viii^	60.51 (19)	N3^ii^—Ba4—Ba2^i^	134.97 (17)
N1—Ba1—Ba4^viii^	103.06 (16)	N2—Ba4—Ba2^i^	47.79 (16)
N2^ii^—Ba1—Ba4^viii^	48.37 (15)	N2^v^—Ba4—Ba2^i^	117.38 (16)
O1^iii^—Ba1—Ba4^viii^	144.23 (18)	O1—Ba4—Ba2^i^	46.44 (17)
O1^iv^—Ba1—Ba4^viii^	118.70 (17)	N1^viii^—Ba4—Ba2^i^	51.70 (15)
Ga1^ii^—Ba1—Ba4^viii^	67.67 (2)	Ga1—Ba4—Ba2^i^	79.68 (2)
Ba2^iv^—Ba1—Ba4^viii^	120.92 (2)	N3^ii^—Ba4—Ba2	136.94 (16)
Ba2^ii^—Ba1—Ba4^viii^	102.089 (19)	N2—Ba4—Ba2	57.57 (17)
N1^i^—Ba1—Ba4^iii^	56.90 (19)	N2^v^—Ba4—Ba2	47.55 (15)
N1—Ba1—Ba4^iii^	59.59 (18)	O1—Ba4—Ba2	56.00 (16)
N2^ii^—Ba1—Ba4^iii^	111.04 (15)	N1^viii^—Ba4—Ba2	120.82 (16)
O1^iii^—Ba1—Ba4^iii^	53.45 (19)	Ga1—Ba4—Ba2	55.77 (2)
O1^iv^—Ba1—Ba4^iii^	135.30 (17)	Ba2^i^—Ba4—Ba2	70.604 (15)
Ga1^ii^—Ba1—Ba4^iii^	77.60 (2)	N3^ii^—Ba4—Ba3^ii^	49.31 (16)
Ba2^iv^—Ba1—Ba4^iii^	108.003 (19)	N2—Ba4—Ba3^ii^	113.90 (17)
Ba2^ii^—Ba1—Ba4^iii^	110.09 (2)	N2^v^—Ba4—Ba3^ii^	142.97 (15)
Ba4^viii^—Ba1—Ba4^iii^	105.50 (2)	O1—Ba4—Ba3^ii^	118.88 (16)
N1^i^—Ba1—Ba2^vii^	47.19 (19)	N1^viii^—Ba4—Ba3^ii^	49.91 (15)
N1—Ba1—Ba2^vii^	112.78 (17)	Ga1—Ba4—Ba3^ii^	121.19 (3)
N2^ii^—Ba1—Ba2^vii^	147.32 (16)	Ba2^i^—Ba4—Ba3^ii^	99.15 (2)
O1^iii^—Ba1—Ba2^vii^	41.16 (18)	Ba2—Ba4—Ba3^ii^	169.46 (2)
O1^iv^—Ba1—Ba2^vii^	116.36 (17)	N3^ii^—Ba4—Ba3^x^	47.62 (16)
Ga1^ii^—Ba1—Ba2^vii^	127.49 (3)	N2—Ba4—Ba3^x^	153.75 (16)
Ba2^iv^—Ba1—Ba2^vii^	63.146 (17)	N2^v^—Ba4—Ba3^x^	79.21 (15)
Ba2^ii^—Ba1—Ba2^vii^	152.86 (2)	O1—Ba4—Ba3^x^	124.02 (16)
Ba4^viii^—Ba1—Ba2^vii^	103.594 (19)	N1^viii^—Ba4—Ba3^x^	106.15 (15)
Ba4^iii^—Ba1—Ba2^vii^	54.148 (15)	Ga1—Ba4—Ba3^x^	123.74 (2)
N1^i^—Ba1—Ba1^i^	42.85 (19)	Ba2^i^—Ba4—Ba3^x^	155.60 (2)
N1—Ba1—Ba1^i^	144.95 (17)	Ba2—Ba4—Ba3^x^	126.28 (2)
N2^ii^—Ba1—Ba1^i^	105.14 (15)	Ba3^ii^—Ba4—Ba3^x^	64.206 (12)
O1^iii^—Ba1—Ba1^i^	92.97 (19)	N3^ii^—Ba4—Ba1^x^	117.25 (17)
O1^iv^—Ba1—Ba1^i^	113.06 (19)	N2—Ba4—Ba1^x^	126.50 (17)
Ga1^ii^—Ba1—Ba1^i^	120.41 (2)	N2^v^—Ba4—Ba1^x^	51.53 (15)
Ba2^iv^—Ba1—Ba1^i^	72.121 (14)	O1—Ba4—Ba1^x^	58.44 (17)
Ba2^ii^—Ba1—Ba1^i^	151.58 (2)	N1^viii^—Ba4—Ba1^x^	109.36 (14)
Ba4^viii^—Ba1—Ba1^i^	57.460 (13)	Ga1—Ba4—Ba1^x^	121.41 (2)
Ba4^iii^—Ba1—Ba1^i^	95.36 (2)	Ba2^i^—Ba4—Ba1^x^	104.86 (2)
Ba2^vii^—Ba1—Ba1^i^	54.518 (17)	Ba2—Ba4—Ba1^x^	70.646 (17)
O1^v^—Ba2—N2^v^	91.9 (3)	Ba3^ii^—Ba4—Ba1^x^	115.65 (2)
O1^v^—Ba2—N1^iii^	84.3 (2)	Ba3^x^—Ba4—Ba1^x^	70.370 (17)
N2^v^—Ba2—N1^iii^	95.3 (2)	N3^ii^—Ba4—Ba1^ii^	116.11 (16)
O1^v^—Ba2—N2	147.5 (2)	N2—Ba4—Ba1^ii^	114.79 (16)
N2^v^—Ba2—N2	92.1 (2)	N2^v^—Ba4—Ba1^ii^	116.15 (16)
N1^iii^—Ba2—N2	63.2 (2)	O1—Ba4—Ba1^ii^	50.26 (16)
O1^v^—Ba2—O1	137.4 (3)	N1^viii^—Ba4—Ba1^ii^	43.92 (14)
N2^v^—Ba2—O1	87.6 (2)	Ga1—Ba4—Ba1^ii^	146.26 (3)
N1^iii^—Ba2—O1	138.1 (2)	Ba2^i^—Ba4—Ba1^ii^	67.004 (17)
N2—Ba2—O1	75.0 (2)	Ba2—Ba4—Ba1^ii^	105.66 (2)
O1^v^—Ba2—O1^vi^	93.5 (3)	Ba3^ii^—Ba4—Ba1^ii^	71.359 (18)
N2^v^—Ba2—O1^vi^	170.4 (2)	Ba3^x^—Ba4—Ba1^ii^	89.987 (19)
N1^iii^—Ba2—O1^vi^	93.1 (2)	Ba1^x^—Ba4—Ba1^ii^	65.465 (13)
N2—Ba2—O1^vi^	87.6 (2)	N3—Ga1—N2	125.3 (3)
O1—Ba2—O1^vi^	83.07 (11)	N3—Ga1—N1^iii^	119.8 (3)
O1^v^—Ba2—Ga1	115.78 (19)	N2—Ga1—N1^iii^	114.6 (4)
N2^v^—Ba2—Ga1	79.89 (17)	N3—Ga1—Ba3^ix^	57.2 (2)
N1^iii^—Ba2—Ga1	34.82 (15)	N2—Ga1—Ba3^ix^	174.9 (2)
N2—Ba2—Ga1	34.00 (14)	N1^iii^—Ga1—Ba3^ix^	62.6 (3)
O1—Ba2—Ga1	106.04 (17)	N3—Ga1—Ba1^iii^	143.7 (2)
O1^vi^—Ba2—Ga1	104.74 (16)	N2—Ga1—Ba1^iii^	65.2 (2)
O1^v^—Ba2—Ba4^v^	57.8 (2)	N1^iii^—Ga1—Ba1^iii^	59.1 (3)
N2^v^—Ba2—Ba4^v^	50.72 (16)	Ba3^ix^—Ga1—Ba1^iii^	110.03 (3)
N1^iii^—Ba2—Ba4^v^	57.98 (17)	N3—Ga1—Ba2	146.1 (2)
N2—Ba2—Ba4^v^	101.71 (14)	N2—Ga1—Ba2	67.8 (2)
O1—Ba2—Ba4^v^	138.28 (17)	N1^iii^—Ga1—Ba2	62.6 (3)
O1^vi^—Ba2—Ba4^v^	138.65 (15)	Ba3^ix^—Ga1—Ba2	112.94 (3)
Ga1—Ba2—Ba4^v^	69.40 (2)	Ba1^iii^—Ga1—Ba2	69.15 (3)
O1^v^—Ba2—Ba4	143.6 (2)	N3—Ga1—Ba3^iii^	62.3 (2)
N2^v^—Ba2—Ba4	51.81 (17)	N2—Ga1—Ba3^iii^	104.3 (2)
N1^iii^—Ba2—Ba4	95.48 (15)	N1^iii^—Ga1—Ba3^iii^	99.1 (3)
N2—Ba2—Ba4	48.63 (13)	Ba3^ix^—Ga1—Ba3^iii^	72.53 (2)
O1—Ba2—Ba4	54.66 (17)	Ba1^iii^—Ga1—Ba3^iii^	81.70 (3)
O1^vi^—Ba2—Ba4	122.74 (17)	Ba2—Ga1—Ba3^iii^	150.54 (4)
Ga1—Ba2—Ba4	61.44 (2)	N3—Ga1—Ba3	50.6 (2)
Ba4^v^—Ba2—Ba4	91.37 (2)	N2—Ga1—Ba3	74.7 (2)
O1^v^—Ba2—Ba1^xi^	94.3 (2)	N1^iii^—Ga1—Ba3	168.6 (3)
N2^v^—Ba2—Ba1^xi^	121.42 (16)	Ba3^ix^—Ga1—Ba3	107.55 (3)
N1^iii^—Ba2—Ba1^xi^	143.29 (17)	Ba1^iii^—Ga1—Ba3	123.58 (3)
N2—Ba2—Ba1^xi^	110.87 (14)	Ba2—Ga1—Ba3	128.70 (3)
O1—Ba2—Ba1^xi^	51.72 (17)	Ba3^iii^—Ga1—Ba3	71.31 (2)
O1^vi^—Ba2—Ba1^xi^	50.26 (15)	N3—Ga1—Ba4	99.0 (2)
Ga1—Ba2—Ba1^xi^	143.49 (3)	N2—Ga1—Ba4	51.9 (2)
Ba4^v^—Ba2—Ba1^xi^	147.07 (2)	N1^iii^—Ga1—Ba4	123.9 (3)
Ba4—Ba2—Ba1^xi^	106.34 (2)	Ba3^ix^—Ga1—Ba4	133.11 (3)
O1^v^—Ba2—Ba1^iii^	106.80 (19)	Ba1^iii^—Ga1—Ba4	110.58 (3)
N2^v^—Ba2—Ba1^iii^	134.13 (17)	Ba2—Ga1—Ba4	62.79 (2)
N1^iii^—Ba2—Ba1^iii^	47.56 (17)	Ba3^iii^—Ga1—Ba4	135.86 (3)
N2—Ba2—Ba1^iii^	50.68 (13)	Ba3—Ga1—Ba4	66.74 (2)
O1—Ba2—Ba1^iii^	103.69 (17)	N3—Ga1—Ba3^v^	47.9 (2)
O1^vi^—Ba2—Ba1^iii^	51.16 (15)	N2—Ga1—Ba3^v^	113.7 (2)
Ga1—Ba2—Ba1^iii^	54.24 (2)	N1^iii^—Ga1—Ba3^v^	113.4 (3)
Ba4^v^—Ba2—Ba1^iii^	105.36 (2)	Ba3^ix^—Ga1—Ba3^v^	71.35 (2)
Ba4—Ba2—Ba1^iii^	99.30 (2)	Ba1^iii^—Ga1—Ba3^v^	167.54 (4)
Ba1^xi^—Ba2—Ba1^iii^	99.012 (17)	Ba2—Ga1—Ba3^v^	98.71 (3)
O1^v^—Ba2—Ba1^ix^	47.36 (18)	Ba3^iii^—Ga1—Ba3^v^	110.13 (3)
N2^v^—Ba2—Ba1^ix^	109.56 (16)	Ba3—Ga1—Ba3^v^	65.886 (19)
N1^iii^—Ba2—Ba1^ix^	41.01 (14)	Ba4—Ga1—Ba3^v^	64.00 (2)
N2—Ba2—Ba1^ix^	101.32 (13)	Ga1^ii^—N1—Ba1^v^	173.9 (5)
O1—Ba2—Ba1^ix^	162.69 (17)	Ga1^ii^—N1—Ba1	84.7 (3)
O1^vi^—Ba2—Ba1^ix^	79.87 (15)	Ba1^v^—N1—Ba1	96.6 (3)
Ga1—Ba2—Ba1^ix^	75.82 (2)	Ga1^ii^—N1—Ba3^v^	81.5 (3)
Ba4^v^—Ba2—Ba1^ix^	58.845 (16)	Ba1^v^—N1—Ba3^v^	101.3 (3)
Ba4—Ba2—Ba1^ix^	134.90 (2)	Ba1—N1—Ba3^v^	137.2 (3)
Ba1^xi^—Ba2—Ba1^ix^	116.853 (17)	Ga1^ii^—N1—Ba2^ii^	82.6 (3)
Ba1^iii^—Ba2—Ba1^ix^	62.918 (10)	Ba1^v^—N1—Ba2^ii^	91.8 (2)
N3^vii^—Ba3—N3^i^	92.5 (2)	Ba1—N1—Ba2^ii^	80.4 (2)
N3^vii^—Ba3—N3	91.05 (3)	Ba3^v^—N1—Ba2^ii^	136.8 (3)
N3^i^—Ba3—N3	157.9 (3)	Ga1^ii^—N1—Ba4^x^	96.7 (3)
N3^vii^—Ba3—N1^i^	71.2 (2)	Ba1^v^—N1—Ba4^x^	79.2 (2)
N3^i^—Ba3—N1^i^	98.9 (2)	Ba1—N1—Ba4^x^	150.1 (3)
N3—Ba3—N1^i^	102.9 (2)	Ba3^v^—N1—Ba4^x^	72.1 (2)
N3^vii^—Ba3—N3^ii^	170.0 (3)	Ba2^ii^—N1—Ba4^x^	70.32 (19)
N3^i^—Ba3—N3^ii^	85.75 (3)	Ga1—N2—Ba2^i^	168.3 (4)
N3—Ba3—N3^ii^	87.0 (2)	Ga1—N2—Ba4	95.7 (3)
N1^i^—Ba3—N3^ii^	118.8 (2)	Ba2^i^—N2—Ba4	81.5 (2)
N3^vii^—Ba3—Ga1^vii^	35.28 (16)	Ga1—N2—Ba4^i^	109.4 (3)
N3^i^—Ba3—Ga1^vii^	97.59 (15)	Ba2^i^—N2—Ba4^i^	80.6 (2)
N3—Ba3—Ga1^vii^	97.89 (16)	Ba4—N2—Ba4^i^	130.0 (3)
N1^i^—Ba3—Ga1^vii^	35.89 (15)	Ga1—N2—Ba1^iii^	79.5 (2)
N3^ii^—Ba3—Ga1^vii^	154.71 (15)	Ba2^i^—N2—Ba1^iii^	96.9 (2)
N3^vii^—Ba3—Ga1^ii^	156.30 (16)	Ba4—N2—Ba1^iii^	148.5 (3)
N3^i^—Ba3—Ga1^ii^	90.65 (16)	Ba4^i^—N2—Ba1^iii^	80.09 (19)
N3—Ba3—Ga1^ii^	94.83 (16)	Ga1—N2—Ba2	78.2 (3)
N1^i^—Ba3—Ga1^ii^	85.14 (15)	Ba2^i^—N2—Ba2	90.1 (2)
N3^ii^—Ba3—Ga1^ii^	33.68 (15)	Ba4—N2—Ba2	73.80 (18)
Ga1^vii^—Ba3—Ga1^ii^	121.03 (4)	Ba4^i^—N2—Ba2	151.9 (3)
N3^vii^—Ba3—Ga1	87.43 (16)	Ba1^iii^—N2—Ba2	74.74 (18)
N3^i^—Ba3—Ga1	126.79 (16)	Ga1—N3—Ba4^iii^	170.9 (4)
N3—Ba3—Ga1	31.61 (16)	Ga1—N3—Ba3^ix^	87.5 (3)
N1^i^—Ba3—Ga1	130.55 (17)	Ba4^iii^—N3—Ba3^ix^	90.0 (2)
N3^ii^—Ba3—Ga1	85.77 (15)	Ga1—N3—Ba3^v^	101.9 (3)
Ga1^vii^—Ba3—Ga1	111.20 (2)	Ba4^iii^—N3—Ba3^v^	87.0 (2)
Ga1^ii^—Ba3—Ga1	109.24 (3)	Ba3^ix^—N3—Ba3^v^	94.4 (2)
N3^vii^—Ba3—Ba4^iii^	88.97 (16)	Ga1—N3—Ba3	97.8 (3)
N3^i^—Ba3—Ba4^iii^	154.98 (16)	Ba4^iii^—N3—Ba3	83.8 (2)
N3—Ba3—Ba4^iii^	46.89 (16)	Ba3^ix^—N3—Ba3	172.1 (3)
N1^i^—Ba3—Ba4^iii^	58.03 (17)	Ba3^v^—N3—Ba3	90.1 (2)
N3^ii^—Ba3—Ba4^iii^	96.78 (14)	Ga1—N3—Ba3^iii^	84.1 (3)
Ga1^vii^—Ba3—Ba4^iii^	69.95 (2)	Ba4^iii^—N3—Ba3^iii^	87.1 (2)
Ga1^ii^—Ba3—Ba4^iii^	78.55 (2)	Ba3^ix^—N3—Ba3^iii^	86.1 (2)
Ga1—Ba3—Ba4^iii^	78.22 (2)	Ba3^v^—N3—Ba3^iii^	174.0 (3)
N3^vii^—Ba3—Ga1^i^	87.80 (16)	Ba3—N3—Ba3^iii^	88.8 (2)
N3^i^—Ba3—Ga1^i^	30.24 (16)	Ba2^i^—O1—Ba1^ii^	91.5 (3)
N3—Ba3—Ga1^i^	128.27 (16)	Ba2^i^—O1—Ba1^xi^	106.8 (3)
N1^i^—Ba3—Ga1^i^	125.19 (17)	Ba1^ii^—O1—Ba1^xi^	139.5 (3)
N3^ii^—Ba3—Ga1^i^	85.74 (14)	Ba2^i^—O1—Ba4	75.8 (2)
Ga1^vii^—Ba3—Ga1^i^	109.63 (3)	Ba1^ii^—O1—Ba4	76.3 (2)
Ga1^ii^—Ba3—Ga1^i^	106.15 (2)	Ba1^xi^—O1—Ba4	142.6 (3)
Ga1—Ba3—Ga1^i^	96.74 (3)	Ba2^i^—O1—Ba2	89.6 (2)
Ba4^iii^—Ba3—Ga1^i^	174.14 (3)	Ba1^ii^—O1—Ba2	144.2 (3)
N3^vii^—Ba3—Ba4^viii^	99.21 (16)	Ba1^xi^—O1—Ba2	73.36 (19)
N3^i^—Ba3—Ba4^viii^	45.37 (16)	Ba4—O1—Ba2	69.3 (2)
N3—Ba3—Ba4^viii^	154.63 (16)	Ba2^i^—O1—Ba2^xii^	86.5 (3)
N1^i^—Ba3—Ba4^viii^	59.70 (17)	Ba1^ii^—O1—Ba2^xii^	72.9 (2)
N3^ii^—Ba3—Ba4^viii^	86.56 (15)	Ba1^xi^—O1—Ba2^xii^	72.5 (2)
Ga1^vii^—Ba3—Ba4^viii^	78.43 (2)	Ba4—O1—Ba2^xii^	143.9 (3)
Ga1^ii^—Ba3—Ba4^viii^	67.05 (2)	Ba2—O1—Ba2^xii^	142.8 (3)
Ga1—Ba3—Ba4^viii^	169.55 (3)		

Symmetry codes: (i) *x*+1/2, *y*, −*z*+1/2; (ii) *x*, −*y*+1/2, *z*+1/2; (iii) *x*, −*y*+1/2, *z*−1/2; (iv) −*x*+1/2, *y*+1/2, *z*; (v) *x*−1/2, *y*, −*z*+1/2; (vi) −*x*+1/2, −*y*, *z*−1/2; (vii) *x*+1/2, −*y*+1/2, −*z*; (viii) *x*+1/2, −*y*+1/2, −*z*+1; (ix) *x*−1/2, −*y*+1/2, −*z*; (x) *x*−1/2, −*y*+1/2, −*z*+1; (xi) −*x*+1/2, *y*−1/2, *z*; (xii) −*x*+1/2, −*y*, *z*+1/2.
